# Cognitive changes associated with chemotherapy in breast cancer: an assessment of social cognition and executive functions in Peruvian patients

**DOI:** 10.1093/oncolo/oyag058

**Published:** 2026-02-25

**Authors:** Sandro Casavilca-Zambrano, Nilton Custodio, Ruddy Liendo-Picoaga, Juan José Contreras Mancilla, Jenny Katherine Bonifacio Mundaca, Rosa Montesinos, Laura Fejerman, Valentina Zavala, Stéphane Bertani, Jorge Honles, Tatiana Vidaurre

**Affiliations:** Facultad de Ciencias de la Salud, Universidad de Huánuco, Huánuco, Peru; Unidad Funcional de Gestión del Banco Nacional de Tumores, Instituto Nacional de Enfermedades Neoplásicas (INEN), Lima, Peru; Instituto Peruano de Neurociencias, Lima, Peru; Unidad Funcional de Gestión del Banco Nacional de Tumores, Instituto Nacional de Enfermedades Neoplásicas (INEN), Lima, Peru; Laboratory of Cell Culture and Immunology Research, Universidad Científica del Sur, Lima, Peru; Unidad Funcional de Gestión del Banco Nacional de Tumores, Instituto Nacional de Enfermedades Neoplásicas (INEN), Lima, Peru; International Joint Laboratory of Molecular Anthropological Oncology (LOAM), INEN–IRD, Lima, Peru; Unidad Funcional de Gestión del Banco Nacional de Tumores, Instituto Nacional de Enfermedades Neoplásicas (INEN), Lima, Peru; International Joint Laboratory of Molecular Anthropological Oncology (LOAM), INEN–IRD, Lima, Peru; Instituto Peruano de Neurociencias, Lima, Peru; UC Davis Comprehensive Cancer Center, University of California Davis, Davis, CA, United States; UC Davis Comprehensive Cancer Center, University of California Davis, Davis, CA, United States; International Joint Laboratory of Molecular Anthropological Oncology (LOAM), INEN–IRD, Lima, Peru; UMR 152 PHARMADEV, Université de Toulouse, Institut de Recherche pour le Développement (IRD), Université Paul Sabatier (UPS), Toulouse, France; UMR 152 PHARMADEV, Université de Toulouse, Institut de Recherche pour le Développement (IRD), Université Paul Sabatier (UPS), Toulouse, France; Departamento de Oncología Médica, Instituto Nacional de Enfermedades Neoplásicas (INEN), Lima, Peru

**Keywords:** ancestry, antineoplastic drugs, breast cancer, chemotherapy-related cognitive impairment, social cognition

## Abstract

**Background:**

Cognitive impairment related to chemotherapy—commonly referred to as “chemo brain”—is a well-documented phenomenon among breast cancer patients. These impairments affect memory, attention, executive function, and social cognition, yet remain understudied in low- and middle-income countries. In Peru, where populations present a high proportion of Amerindian ancestry and distinct sociocultural factors, evidence is scarce.

**Methods:**

We conducted a longitudinal study of 143 Peruvian women aged 28-64 years, newly diagnosed with early-stage breast cancer and naïve to chemotherapy, treated at the National Institute of Neoplastic Diseases (INEN) in Lima. Cognitive function was assessed using the Addenbrooke’s Cognitive Examination (ACE), the INECO Frontal Screening Test (IFS), and a Facial Emotion Recognition (FER) task to evaluate social cognition. Baseline tests were performed before the start of treatment for each patient, and post-treatment tests were performed every 3 months. Global genetic ancestry was estimated using the ADMIXTURE algorithm based on the Affymetrix Precision Medicine Research Array.

**Results:**

Native American ancestry accounted for 77.8% of the study population. Post-chemotherapy assessments revealed cognitive impairment in 21% of patients based on FER, 15% on ACE, and 12% on IFS. Higher educational attainment was associated with better cognitive performance across all domains.

**Conclusion:**

Chemotherapy was associated with measurable cognitive decline in a subset of Peruvian breast cancer patients. Brief and culturally adaptable tools such as the FER test offer a promising approach for routine cognitive screening in oncology settings, particularly in resource-limited contexts. Incorporating these assessments into standard care may facilitate early detection and more personalized supportive interventions.

Implications for Practice This study underscores the need to integrate culturally adapted cognitive and emotional screening tools into the routine assessment of women undergoing chemotherapy for breast cancer, particularly within underserved populations. Early recognition of cognitive changes can inform individualized therapeutic strategies and supportive interventions aimed at preserving quality of life, promoting emotional well-being, and enhancing treatment adherence in clinical practice.

## Introduction

Cognitive ability, shaped by evolution, allows us to perceive, process, and interpret information from our environment. It is not learned but a function of our sensory and central nervous systems, making the brain a predictive machine that reduces uncertainty by interpreting stimuli.^1–3^ Cognitive changes, often manifesting as difficulties in attention, memory, concentration, and executive functioning, have been reported in 16% to 75% of women undergoing chemotherapy for breast cancer.^4–8^

In Peru, breast cancer is the most prevalent malignancy among women and the second leading cause of cancer-related mortality.^9^^,^^10^ The Peruvian population is characterized by high Amerindian ancestry, which has been linked to unique epidemiological patterns in disease susceptibility and outcomes.^11–19^ According to GLOBOCAN, an estimated 7,797 new breast cancer cases were projected for Peru by 2022, making it the most common cancer among women, predominantly affecting the economically active population. The mean age of onset is 49 years, a decade earlier than that observed in Europe and the United States, with a higher incidence of triple-negative breast cancer, which often necessitates more aggressive chemotherapy.^14–16^^,^^20–22^

With the aging population, both degenerative diseases such as dementia and breast cancer are on the rise, highlighting the need for studies that explore the prevalence of cognitive impairment in women diagnosed with breast cancer and assess its impact on this population.^23^^,^^24^ Our study aimed to determine the prevalence of cognitive impairment using brief psychological tests, including the Addenbrooke’s Cognitive Examination (ACE) and the INECO Frontal Screening (IFS) test, to evaluate general cognition and executive functions. Additionally, we sought to assess Facial Emotion Recognition (FER) as an objective, accessible tool for evaluating social cognition.^25–33^ The INECO Frontal Screening (IFS), Addenbrooke’s Cognitive Examination (ACE), and Facial Emotion Recognition (FER) tests instead of the tools recommended by the International Cognition and Cancer Task Force (ICCTF) because they offered broader, more integrative insights into the cognitive and emotional status of our participants. Specifically, we worked with Peruvian women diagnosed with breast cancer, most of whom had incomplete formal education. While the ICCTF tools (such as the Trail Making Test, COWAT, and HVLT-R) are widely validated for assessing isolated cognitive domains, they often assume a level of education or cultural familiarity that may limit their applicability in lower-education settings. In contrast, the IFS allowed us to explore executive functions in a more comprehensive and accessible way, the ACE provided a global cognitive screening tailored to Spanish-speaking populations, and the FER test added a valuable dimension by addressing social and emotional processing—an area not covered by the ICCTF battery. Additionally, unlike many ICCTF-based studies that rely on comparisons with a control group, our study did not include a control group, making it even more important to use screening tools capable of capturing a broad and meaningful cognitive profile within the patient group alone. Together, these instruments helped ensure that our cognitive assessments were not only scientifically robust but also culturally and contextually appropriate, capturing the realities and needs of the women we aimed to study.

## Materials and methods

### Patients’ sampling and study design

This longitudinal study, conducted at the National Institute of Neoplastic Diseases (INEN) in Lima, Peru, focuses on women with early-stage breast cancer. It included 143 female patients aged 28-64 years, diagnosed with breast cancer who had not received prior chemotherapy. To ensure eligibility, participants underwent a Beck Depression Inventory-II test; those with a score below 14 or symptoms of depression were excluded. Blood tests for B12, TSH, T3, and T4 were also performed, and patients with results outside the normal range were excluded.

The COVID-19 pandemic limited the recruitment of new patients to complete the number of patients scheduled for the study. The patients involved had to meet inclusion criteria such as women older than 18 years diagnosed with breast cancer, not having received chemotherapy, having at least 4 years of elementary education; and exclusion criteria such as: presence of metastasis, some type of neurological disorder (Alzheimer’s disease, vascular dementia, frontotemporal dementia, epilepsy, febrile seizures, Parkinson’s disease, and others), any type of autoimmune disease, pregnant patients, if receiving additional treatment, patients with vascular cognitive disorder based on assessment of vascular risk factors (hypertension, diabetes mellitus, hyperlipidemia, obesity, and others), and modified Hachinski index; and elimination criteria such as: insufficient biological material, incomplete cognitive function assessment.

All participants provided informed consent, and the study was approved by the INEN Ethics Committee. Clinical and sociodemographic information was compiled into a database. Blood samples were collected by INEN-trained personnel. The study involved baseline cognitive and psychological assessments, with follow-up cognitive testing conducted quarterly at 3, 6, and 9 months after the initiation of chemotherapy. The type of chemotherapy received by each patient was also recorded.

### Psychological brief cognitive tests

The psychological assessment, conducted by a psychologist, included the IFS test, the Facial Emotion Recognition (FER) test, and the ACE test. These tests were used to evaluate executive functions, social cognition, and general cognition, respectively and were administered in their validated Peruvian versions.^25–28^

ACE assessed 6 cognitive domains with a maximum score of 100 points: orientation (10 points), attention (8 points), memory (35 points), verbal fluency (14 points), language (28 points), and visual-spatial skills (5 points). Additionally, ACE included components of the Mini-Mental State Examination (MMSE), contributing 30 points to the overall score. While the MMSE allocated 3/30 points to memory function, ACE assigned 35/100 points. The Peruvian study identified a cut-off score of 86 out of 100 as most effective for detecting dementia, demonstrating high sensitivity and specificity. This cut-off point also ensures that dementia classification is consistent regardless of sex, age, or years of education.^26^

IFS is a concise cognitive assessment tool used to evaluate executive functions, consisting of 8 subtests. These include motor programming (3 points), conflicting instructions (3 points), inhibitory motor control (3 points), digit counting in reverse order (6 points), verbal working memory (2 points), spatial working memory (4 points), capacity for abstraction (3 points), and verbal inhibitory control (6 points). With a maximum total score of 30 points, IFS can be administered and scored in approximately 10 minutes. A score of 23 or below indicated executive and social impairment. The Peruvian version of IFS has demonstrated high efficacy, with a sensitivity of 94.12% and a specificity of 94.2%, surpassing the Frontal Assessment Battery in its ability to detect cognitive impairments.^29^

FER involved identifying facial expressions that convey basic emotions such as fear, happiness, and disgust. Basic human emotions are categorized into 6 types: anger, disgust, fear, happiness, sadness, and surprise, in addition to neutral expressions.^30–35^ In our evaluation, 7 facial expressions were assessed: happiness, surprise, neutral, sadness, fear, disgust, and anger. These expressions are presented in a set of 35 images used for the test.

### Chemotherapy schemes

All patients received the same chemotherapy regimen, which included hormonotherapy, anthracyclines, and taxanes, with some variations based on the pathological diagnosis and immunohistochemistry approach related to the type of breast cancer. The standard chemotherapy regimen consisted of doxorubicin hydrochloride (Adriamycin) and cyclophosphamide, followed by paclitaxel (Taxol), specifically administered as Adriamycin + Cyclophosphamide (AC) followed by Paclitaxel (Table S1).

### Genotyping and quality control (QC)

Genotyped data were obtained with the Affymetrix Precision Medicine Research Array (PMRA) Chip, and quality control of the whole genome genotype was performed in PLINK v1.933. Markers from sex chromosomes were excluded; SNPs with more than 2% absence in the data, Hardy–Weinberg equilibrium at a *P*-value of <5 × 10^−5^ and alleles with a minor allele frequency (MAF) below 5% were excluded. Individuals with more than 5% loss of genotyped information were excluded. The search for genetically related individuals was performed using KING v2.2.5 to be removed from the study (no related participants >12.5% were found). After performing the QC, palindromic variants were eliminated.

### Genetic ancestry and principal component analysis (PCA)

Individual global genetic ancestry was estimated using ADMIXTURE, ADMIXTURE continental ancestry estimates obtained in unsupervised analysis, assuming *K* = 4. Genotyped data were pruned using PLINK v1.933 and merged with data from the 1000 Genomes Project with admixed Americans, Europeans, east Asians, and African populations. PCA was performed in unrelated individuals to estimate the genetic structure of the Peruvian population under study (135 participants).

### Identification of genetic polymorphisms for APOE and TREM2 genes

R Studio and PLINK v1.933 were used to select the polymorphisms that were studied in the APOE and TREM2 genes from the genotyped data (without QC, simply MAF). The genetic information that was discovered for each person in the study was combined with their phenotype (as measured by the ACE, IFS, and FER psychological tests) and their ancestry values for each continental group. Data on sociodemographic characteristics were listed among the covariates.

### Statistical analysis

To evaluate changes in cognitive performance over time and in accordance with the study design, a multivariate analysis was performed; a linear mixed model of repeated measures was chosen. 4 cut-off points were established for each evaluation (E0, E1, E2, and E3); as follows: E0—for Baseline scores (prior to the start of chemotherapy), E1—for Evaluation 1 (3 months after starting treatment), E2—for Evaluation 2 (6 months after starting treatment) and E3—for Evaluation 3 (9 months started treatment). To fit the model, fixed and random effects were considered. The fixed effect variables were evaluation 0 (Baseline), age, level of education, number of normal pregnancies, number of abortions, and menopause. Each patient was considered as a random variable and as a dependent variable, the scores of each evaluation in the sections (Assessment 1, 2, and 3); corresponding to 3, 6, and 9 months of starting the treatment to find the minimum clinically important difference, the patients who presented sustained alterations in the E2 and E3 controls of the study were evaluated.^36^^,^^37^ Chemotherapy regimens were included as a time interaction covariate. In addition, the Toeplitz structure matrix and an autoregressive structure model (AR1) were used. The Akaike Information Criterion was used as a reference for estimating variance parameters for each analysis. A *P*-value <.05 was considered for a statistically significant difference or relationship. The PostgreSQL 12, SPSS 27, and R Studio 1.3.1093 packages were used.

## Results

### Sociodemographic and clinical characteristics

The mean age at diagnosis of invasive breast cancer was 48.3 years (SD = 8.98). The average age at menarche was 13 ± 1.7 years, and the mean age at menopause was 46.5 ± 4.9 years. Among the participants, 63 women (50.8%) were premenopausal. The majority, 72 patients (58.1%), resided in the Lima region, while 52 (41.9%) lived in other regions of the country.

Regarding educational level, 91 women (73.4%) had completed secondary education, whereas 33 (26.6%) had not; the average number of years of schooling was 11.4. Marital status distribution showed that 31 women (25.0%) were cohabitants, 42 (33.9%) were married, 50 (40.3%) were single (including separated, divorced, or widowed), and one participant (0.8%) did not report this information. Employment status indicated that 5 women (4%) were employed, 54 (43.5%) were not working (including those previously employed), 19 (15.3%) were self-employed, and 46 (37.1%) were unemployed.

In terms of reproductive history, 16 women (12.9%) had never been pregnant, 15 (12.1%) reported one pregnancy, 26 (21%) had 2 pregnancies, and 67 women (54%) had more than 2 pregnancies (Table 1).

**Table 1.** General and sociodemographic characteristics of patients (*n* = 124).

	Means (SD)
**Continuous variables**	
** Age, years**	48.3 (8.9)
** Age at menarche, years**	13.0 (1.7)
** Age at menopause (*N* = 61), years**	46.5 (4.9)
** Years of education**	11.4 (3.3)
**Categorical variables**	
** Place of residence**	
** Lima Region**	72 (58.1)
** Other regions**	52 (41.9)
**Education level**	
** Completed secondary**	91 (73.4)
** Incomplete secondary**	33 (26.6)
**Marital status**	
** Cohabitants**	31 (25.0)
** Married**	42 (33.9)
** Single^a^**	50 (40.3)
** No data available**	1 (0.8)
**Employment situation**	
** Employees**	5 (4.0)
** No current activity**	54 (43.5)
** Independent**	19 (15.3)
** Unemployed**	46 (37.1)
**Pregnancies**	
** 0**	16 (12.9)
** 1**	15 (12.1)
** 2**	26 (21.0)
** >2**	67 (54.0)
**Abortions**	
** 0**	72 (58.1)
** 1**	33 (26.6)
** 2**	16 (12.9)
** 3**	2 (1.6)
** 4**	1 (0.8)

a Divorced, widowed, and separated people are included.

### Cognitive performance

Patients received an average of 10.8 chemotherapy cycles. The most commonly administered regimen was Adriamycin combined with cyclophosphamide followed by paclitaxel (AC + Paclitaxel), received by 71 patients (57.3%). Those with triple-negative breast cancer received adjuvant capecitabine.

Cognitive function was assessed using 3 instruments: the IFS for executive function, ACE for global cognition, and the FER for social cognition. According to these assessments, the FER test identified impairments in 21% of patients, the IFS test in 12%, and the ACE test in 15% (Table 2).

**Table 2.** Total of patients that showed cognitive performance declining from E1 to E3 (*n* = 19).

Test	Evaluations				
	E0	E1	E2		E3
**FER**	25	30	27		21
	27	29	26		17
	15	26	22		17
	24	31	30		29
	25	31	28		25
	26	30	28		25
	26	33	32		12
	23.38 ± 4.1	29.75 ± 2.2	27.25 ± 3.2		20.75 ± 5.9
**Percent with decrease/impairment**			21% IC 95% (7.3-34.7)		
**IFS**	18	25	22		19
	14	20	19		16
	25	25	24		20
	14	26	23		18
	17.75 ± 5.2	24 ± 2.7	22 ± 2.2		18.25 ± 1.7
**Percent with decrease/impairment**			12% IC 95% (1.1-22.9)		
**ACE**	90	92	91		89
	83	94	93		92
	89	95	94		90
	86	95	92		91
	97	97	96		76
	90 ± 5.2	94.6 ± 1.8	9.2 ± 1.9		87.6 ± 6.6
**Percent with decrease/impairment**				15% IC 95% (3-27)	

The emotion “fear” was the most difficult to recognize, whereas “happiness,” “surprise,” and “neutral” were recognized more accurately. Lower FER scores were observed in patients with poorer executive and cognitive performance, both before and after chemotherapy (Table 3). Higher IFS and ACE scores were associated with better emotion recognition performance. Additionally, patients with at least secondary education showed better cognitive outcomes across domains including facial recognition, orientation, attention, concentration, memory, and language.

**Table 3.** A summary of the emotion recognition scores (mean ± SD).

	Evaluations (*n* = 124)			
	E0	E1	E2	E3
**Fear score**	1.84 ± 1.28	2.15 ± 1.28	2.46 ± 1.28	2 ± 1.55
**Disgust score**	3.28 ± 1.48	3.58 ± 1.4	3.77 ± 1.24	3.29 ± 1.32
**Anger score**	3.48 ± 1.09	3.95 ± 0.93	4.25 ± 0.85	3.34 ± 1.39
**Sadness score**	3.74 ± 1.14	4.10 ± 0.97	4.27 ± 0.93	4.09 ± 1.12
**Neutral score**	3.89 ± 1.49	4.28 ± 1.14	4.26 ± 1.19	4.06 ± 1.61
**Surprise score**	4.11 ± 1.17	4.64 ± 0.64	4.35 ± 1.09	4.37 ± 1.19
**Happiness score**	4.9 ± 0.36	4.99 ± 0.09	4.99 ± 0.11	4.94 ± 0.34
**Total score**	25.23 ± 4.29	27.68 ± 3.23	28.34 ± 3.83	26.09 ± 5.3

**Figure 1.**
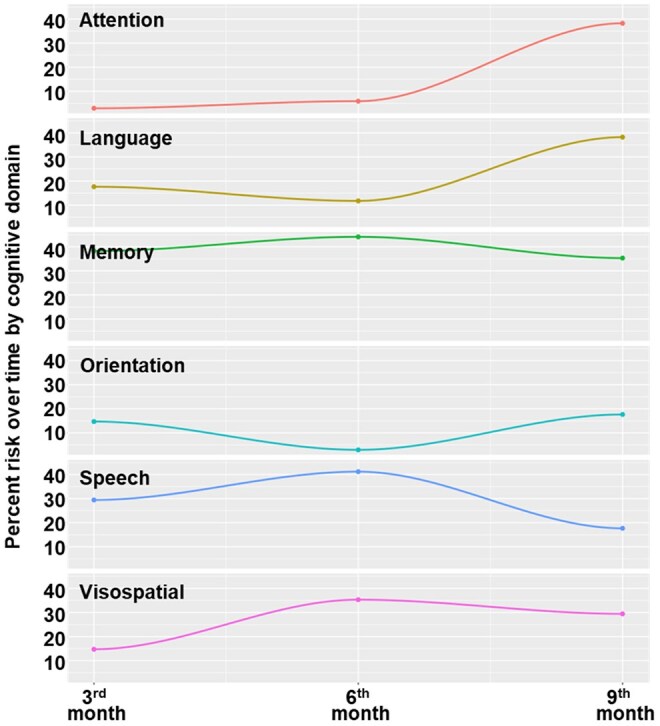
Chemotherapy-related cognitive changes over time based on risk behavior by domain.

Notably, attention and language domains showed greater risk of impairment by the sixth month of chemotherapy. Visuospatial ability, already affected at 6 months, declined further by the ninth month of treatment (Figure 1, Table 4).

**Table 4.** Changes in cognitive ability after chemotherapy depending on the domain (*n* = 124).

Domain	Third month (%)	Sixth month (%)	Ninth month (%)
**Orientation**	14.71	2.94	17.64
**Attention**	2.94	5.88	38.23
**Memory**	38.24	44.12	35.29
**Verbal**	29.41	41.18	17.65
**Language**	17.65	11.76	38.24
**Visuospatial**	14.71	35.29	29.41

### Genetic and ancestry findings

Ancestry analysis was conducted in 135 individuals. Amerindian ancestry was predominant, representing 77.8% of the cohort, followed by European ancestry (15.7%) and African ancestry (mean 5.8%). The average African genetic ancestry observed was higher than that reported in previous studies of Peruvian patients with breast cancer.^15^ The detailed distribution of ancestry components by individual is available in the Supplementary Material (Table S2).

## Discussion

Chemotherapy treatment has been associated with alterations in cognitive performance, particularly affecting executive functions and emotion recognition, thus revealing impairments in social cognition processes. In our study, executive functions were evaluated using the FER, ACE, and IFS tests. The FER test identified decreased cognitive performance in 21% of patients, while the ACE and IFS tests showed declines in 15% and 12% of cases, respectively. These findings are consistent with previous reports indicating a measurable impact of chemotherapy on executive function domains, such as attention, working memory, and verbal fluency.^1^^,^^38^

Notably, better emotion recognition performance was associated with higher ACE and IFS scores, supporting the close interaction between cognitive and emotional processing. Fear—an emotion strongly associated with the temporal lobes and amygdala (Theory of Mind)—was the most difficult emotion for patients undergoing chemotherapy to recognize.^1^^,^^38^ This aligns with existing literature highlighting the amygdala’s crucial role in emotion and social behavior. Studies in individuals with amygdala lesions have demonstrated impaired recognition of emotional facial expressions, particularly fear, reinforcing its importance in emotional processing.^39^^,^^40^

Research using animal models and neuroimaging has shown that chemotherapy can directly affect cognitive function, especially in frontal regions responsible for executive and memory-related tasks. Emotion recognition via facial expression, a universal phenomenon widely studied in psychology, has been explored since Darwin’s evolutionary theory of 1868, which proposed that such expressions serve an adaptive role in communication and survival.^41^ According to this theory, facial expressions are biologically encoded and evolved through natural selection to facilitate social interaction and understanding.

In our analysis, emotion recognition scores were consistently lower among patients with executive and cognitive impairments, across all evaluations (E0, E1, E2, and E3) (Table 2). Moreover, patients with a high school education or higher exhibited better performance in cognitive domains such as facial recognition, orientation, attention, memory, and language (Table 4). These findings suggest the influence of cognitive reserve, consistent with prior observations that lower education levels are associated with higher vulnerability to cognitive dysfunction and increased mortality.^42^

Specifically, IFS testing revealed that 12% of patients exhibited executive function impairments, affecting abilities such as inhibitory control, attention regulation, and flexible thinking.^43–46^ In our study, attention and language domains were particularly affected at the sixth-month chemotherapy follow-up (Table 4). A decrease in visuospatial ability was observed at this time point as well, dropping from 35.29 to 29.41 by the ninth-month follow-up.

The identification of predictive factors for cognitive performance in breast cancer patients remains challenging. There is a clear need for brief, user-friendly diagnostic tools to assess executive functions and social cognition.^47^^,^^48^ An ideal screening instrument would be concise, require minimal materials, be easy to score, and demonstrate high sensitivity and specificity for detecting cognitive impairment.^49^

Interestingly, several studies have reported that cognitive deficits may be present even before the start of chemotherapy, suggesting a possible baseline vulnerability. These deficits may be influenced by cancer-related biological changes or shared risk factors for both cancer and cognitive decline.^50^^,^^51^ Longitudinal studies have shown that 20%-30% of patients exhibit cognitive impairments prior to treatment initiation.^52^ In line with this, Jansen et al. (2011) found that 23% of 71 breast cancer patients had cognitive dysfunction before starting chemotherapy.^53^

The most frequently used chemotherapy protocol in our cohort was a combination of doxorubicin hydrochloride (Adriamycin) and cyclophosphamide followed by paclitaxel (Taxol), administered to 71 patients (57.3%). This regimen (AC + Paclitaxel) has previously been associated with neurotoxic effects. Moreover, when taxanes are combined with hormonal therapy, cognitive decline appears to intensify, although the duration of these effects remains unclear.^53^

The mechanisms underlying chemotherapy-induced cognitive impairment are not yet fully understood, though changes in the central nervous system are known to be heterogeneous. Several hypotheses have been proposed, including toxicity to non-tumor cells, microvascular damage, hormonal disruption, and immune activation via cytokines such as TNF-α.^54^^,^^55^ Additionally, genetic factors such as TREM2 and APOE may modulate microglial activity and influence cognitive outcomes. The involvement of these genes in “chemo brain” and their interaction with ancestry-specific polymorphisms remains an area of active investigation.^56^^,^^57^

It remains unclear how genetic polymorphisms influence the risk of cognitive decline in breast cancer patients.^58–69^ Given Peru’s diverse genetic makeup, the prevalence and impact of such polymorphisms may differ significantly compared to other populations.^13^^,^^24^^,^^70–73^ Ancestry plays a key role in the frequency of risk alleles. For example, the APOE ε4 allele has a lower frequency in Peruvians (4.6%) compared to Caucasian populations, and TREM2 variants have not been studied in cognitively impaired Peruvian individuals. Marca et al. identified the APOE ε4 allele in 4.6% of cognitively normal Peruvians.^25^^,^^73^ Genetic studies in Peruvians have shown a predominant Quechua ancestral component in central regions, with Andean-Piedmont subcomponents in the north and a smaller Aymara influence in the south.^74^^,^^75^

In our sample, Amerindian ancestry comprised 77.8%, and the proportion of African ancestry (5.8%) was higher than in other Peruvian studies of breast cancer patients.^15^ This population structure presents a unique opportunity to evaluate how Amerindian ancestry and related genetic polymorphisms may contribute to cognitive decline and dementia risk.^14^^,^^16^^,^^20^ Accurate assessment of cognitive impairment and the implementation of tailored clinical protocols will be essential for understanding the underlying biological mechanisms and identifying potential molecular markers associated with cognitive deterioration.^76^

## Conclusions

The implementation of brief, accessible, and objective cognitive tests that provides independent evaluation of educational levels is essential for assessing patients undergoing chemotherapy in standard-of-care settings. The FER test is a promising tool for this purpose, as it is easy to implement and does not require specialized personnel for administration.

Additionally, studying cognitive impairment caused by cancer chemotherapy is crucial to identify genes and polymorphisms associated with this condition in the Peruvian population, which has a high Amerindian ancestry and has been underrepresented in cancer research. Such research will enhance our understanding of risk factors and enable the development of tailored treatment options.

## Supplementary Material

oyag058_Supplementary_Data

## Data Availability

The authors confirm that the data supporting the findings of this study are available within the article.

## References

[1] Tirapu-Ustárroz J , Pérez-Sayes G, Erekatxo-Bilbao M. Pelegrín-valero C. ¿Qué es la teoría de la mente? Rev Neurol. 2007;44:479-489. 17455162

[2] Bubic A , Yves von Cramon D, Schubotz RI. Prediction, cognition and the brain. Vol. 4, Frontiers in Human Neuroscience. Frontiers Media S. A.; 2010. 10.3389/fnhum.2010.00025 PMC2904053 20631856

[3] De Ridder D , Verplaetse J, Vanneste S. The predictive brain and the “free will” illusion. Front Psychol. 2013;4:10.3389/fpsyg.2013.00131 PMC3639403 23641219

[4] Argyriou AA , Assimakopoulos K, Iconomou G, Giannakopoulou F, Kalofonos HP. Either called “chemobrain” or “chemofog,” the long-term chemotherapy-Induced cognitive decline in cancer survivors is real. J Pain Symptom Manage. 2011;41:126-139. 20832978 10.1016/j.jpainsymman.2010.04.021

[5] Ibrahim EY , Domenicano I, Nyhan K, et al Cognitive effects and depression associated with taxane-based chemotherapy in breast cancer survivors: a meta-analysis. Front Oncol. 2021;11:642382. 33996556 10.3389/fonc.2021.642382 PMC8121254

[6] Vardy J , Rourke S, Tannock IF. Evaluation of cognitive function associated with chemotherapy: a review of published studies and recommendations for future research. J Clin Oncol. 2007;25:2455-2463. 17485710 10.1200/JCO.2006.08.1604

[7] Breckenridge LM , Bruns GL, Todd BL, Feuerstein M. Cognitive limitations associated with tamoxifen and aromatase inhibitors in employed breast cancer survivors. Psychooncology. 2012;21:43-53. 20967847 10.1002/pon.1860

[8] Hermelink K. Chemotherapy and cognitive function in breast cancer patients: the so-called chemo brain. J Natl Cancer Inst Monogr. 2015;2015:67-69. 26063891 10.1093/jncimonographs/lgv009

[9] Instituto Nacional de Enfermedades Neoplásicas. Registro de Cáncer de Lima Metropolitana. Incidencia y mortalidad 2013—2015. 2021.

[10] Ramos Muñoz WC , Guerrero Ramírez NN. Análisis de la situación del cáncer en el Perú, 2018. Ministerio de Salud del Perú; 2020.

[11] Harris DN , Song W, Shetty AC, et al Evolutionary genomic dynamics of Peruvians before, during, and after the Inca Empire. Proc Natl Acad Sci USA. 2018;115:E6526-E6535. 29946025 10.1073/pnas.1720798115 PMC6048481

[12] Sandoval JR , Salazar-Granara A, Acosta O, et al Tracing the genomic ancestry of Peruvians reveals a major legacy of pre-Columbian ancestors. J Hum Genet. 2013;58:627-634. 23863748 10.1038/jhg.2013.73

[13] Asgari S , Luo Y, Huang C-C, et al Higher native Peruvian genetic ancestry proportion is associated with tuberculosis progression risk. Cell Genom. 2022;2:100151. 35873671 10.1016/j.xgen.2022.100151 PMC9306274

[14] Zavala VA , Casavilca-Zambrano S, Navarro-Vásquez J, et al Association between ancestry-specific 6q25 variants and breast cancer subtypes in Peruvian women. Cancer Epidemiol Biomarkers Prev. 2022;31:1602-1609. 35654312 10.1158/1055-9965.EPI-22-0069 PMC9662925

[15] Marker KM , Zavala VA, Vidaurre T, et al Human epidermal growth factor receptor 2–positive breast cancer is associated with indigenous American ancestry in Latin American women. Cancer Res. 2020;80:1893-1901. 32245796 10.1158/0008-5472.CAN-19-3659 PMC7202960

[16] Tamayo LI , Vidaurre T, Navarro Vásquez J, et al Breast cancer subtype and survival among Indigenous American women in Peru. PLoS One. 2018;13:e0201287. 30183706 10.1371/journal.pone.0201287 PMC6124707

[17] Shieh Y , Fejerman L, Lott PC, et al A polygenic risk score for breast cancer in U.S. Latinas and Latin-American women. bioRxiv. 2019. 10.1093/jnci/djz174 PMC7301155 31553449

[18] Marchio A , Cerapio JP, Ruiz E, et al Early-onset liver cancer in South America associates with low hepatitis B virus DNA burden. Sci Rep. 2018;8:12031-12014. 30104677 10.1038/s41598-018-30229-8 PMC6089985

[19] Cerapio JP , Marchio A, Cano L, et al Global DNA hypermethylation pattern and unique gene expression signature in liver cancer from patients with Indigenous American ancestry. Oncotarget. 2021;12:475-492. 33747361 10.18632/oncotarget.27890 PMC7939527

[20] Lynce F , Graves KD, Jandorf L, et al Genomic disparities in breast cancer among latinas. Cancer Control. 2016;23:359-372. 10.1177/107327481602300407 27842325 PMC5160045

[21] Stern MC , Fejerman L, Das R, et al Variability in cancer risk and outcomes within US Latinos by national origin and genetic ancestry. Curr Epidemiol Rep. 2016;3:181-190. 10.1007/s40471-016-0083-7 27547694 PMC4978756

[22] Shieh Y , Fejerman L, Lott PC, et al A polygenic risk score for breast cancer in US Latinas and Latin American women. J Natl Cancer Inst. 2020;112:590-598. 31553449 10.1093/jnci/djz174 PMC7301155

[23] Anuj S , Bhaniani A, Wareham N, et al Cognitive function in a general population of men and women: a cross sectional study in the European Investigation of Cancer–Norfolk cohort (EPIC-Norfolk). BMC Geriatr. 2015;14:142. 10.1186/1471-2318-14-142 PMC4349767 25527303

[24] Custodio N , Wheelock A, Thumala D, Slachevsky A. Dementia in Latin America: epidemiological evidence and implications for public policy. Front Aging Neurosci. 2017;9:221-211. http://journal.frontiersin.org/article/10.3389/fnagi.2017.00221/full 28751861 10.3389/fnagi.2017.00221 PMC5508025

[25] Custodio N , Alva-Diaz C, Becerra-Becerra Y, et al Rendimiento en pruebas cognitivas breves, DE adultos mayores con demencia en estadios avanzados, residentes de una comunidad urbana de Lima, perú. Rev Peru Med Exp Salud Publica. 2016;33:662-669. 28327834 10.17843/rpmesp.2016.334.2549

[26] Custodio N , Lira D, Montesinos R, Gleichgerrcht E. Utilidad del Addenbrooke’s cognitive examination versión en español en pacientes peruanos con enfermedad de Alzheimer y demencia frontotemporal. Rev Arg de Psiquiat. 2012;XXIII:165-172. 23145370

[27] Custodio N , Duque L, Montesinos R, Alva-Diaz C, Mellado M, Slachevsky A. Systematic review of the diagnostic validity of brief cognitive screenings for early dementia detection in Spanish-speaking adults in Latin America. Front Aging Neurosci. 2020;12:270-213. 33101004 10.3389/fnagi.2020.00270 PMC7500065

[28] Custodio N , Montesinos R, Lira D, Bendezu L, Herrera E. Validation of a Peruvian version of the Memory Alteration Test administered to people with amnestic mild cognitive impairment and early-stage Alzheimer’s disease in Lima, Peru. Alzheimer’s Dement. 2013;9:

[29] Custodio N , Herrera-Perez E, Lira D, et al Evaluation of the INECO frontal screening and the frontal assessment battery in Peruvian patients with Alzheimer’s disease and behavioral variant frontotemporal dementia. eNeurologicalSci. 2016;5:25-29. 29430554 10.1016/j.ensci.2016.11.001 PMC5803087

[30] Santoso BE , Kusuma GP. Facial emotion recognition on FER2013 using vggspinalnet. J Theor Appl Inf Technol. 2022; 100:2088-2102.

[31] Ekman P. An argument for basic emotions. Cogn Emot. 1992; 6:169-200.

[32] Tarnowski P , Kołodziej M, Majkowski A, Rak RJ. Emotion recognition using facial expressions. In: Procedia Computer Science. Elsevier B.V.; 2017:1175-1184.

[33] Chen M , Cheng J, Zhang Z, Li Y, Zhang Y. Facial expression recognition method combined with attention mechanism. Mobile Inform Syst. 2021;2021:1.

[34] Adolphs R, Damasio H, Tranel D, Damasio AR. Cortical systems for the recognition of emotion in facial expressions. J Neurosci. 1996;16:7678–7687. 8922424 10.1523/JNEUROSCI.16-23-07678.1996 PMC6579085

[35] Lee SC , Liu CC, Kuo CJ, Hsueh IP, Hsieh CL. Sensitivity and specificity of a facial emotion recognition test in classifying patients with schizophrenia. J Affect Disord. 2020;275:224-229. 32734912 10.1016/j.jad.2020.07.003

[36] de Vet HCW , Ostelo RWJG, Terwee CB, et al Minimally important change determined by a visual method integrating an anchor-based and a distribution-based approach. Qual Life Res. 2007;16:131-142. 17033901 10.1007/s11136-006-9109-9 PMC2778628

[37] Cheung YT , Foo YL, Shwe M, et al Minimal clinically important difference (MCID) for the functional assessment of cancer therapy: cognitive function (FACT-Cog) in breast cancer patients. J Clin Epidemiol. 2014;67:811-820. 24656406 10.1016/j.jclinepi.2013.12.011

[38] De León JMRS , Blázquez MÁF. Arquitecturas cognitivas y cerebro: Hacia una teoría unificada de la cognición. Int J Psychol Res (Medellin). 2011;4:38-47.

[39] Tirapu-Ustárroz J , Pérez-Sayes G, Erekatxo-Bilbao M, Pelegrín-Valero C. Qué es la teoría de la mente? Rev Neurol (Paris). 2007;44:479-489. http://ps.carlos.cortes.googlepages.com/TEORADELAMENTE.pdf 17455162

[40] Calder AJ , Young AW, Rowland D, Perrett DI, Hodges JR, Etcoff NL. Facial emotion recognition after bilateral amygdala damage: differentially severe impairment of fear. Cogn Neuropsychol. 1996;13:699-745.

[41] Krause FC , Linardatos E, Fresco DM, Moore MT. Facial emotion recognition in major depressive disorder: a meta-analytic review. J Affect Disord. 2021;293:320-328. 34229285 10.1016/j.jad.2021.06.053 PMC8457509

[42] Hayat SA , Luben R, Dalzell N, et al Understanding the relationship between cognition and death: a within cohort examination of cognitive measures and mortality. Eur J Epidemiol. 2018;33:1049-1062. 10.1007/s10654-018-0439-z 30203336 PMC6208995

[43] Diamond A. Executive functions. Annu Rev Psychol. 2013;64:135-168. 23020641 10.1146/annurev-psych-113011-143750 PMC4084861

[44] Holohan KN , Von Ah D, McDonald BC, Saykin AJ. Neuroimaging, cancer, and cognition: state of the knowledge. Semin Oncol Nurs. 2013;29:280-287. 24183159 10.1016/j.soncn.2013.08.008 PMC3821968

[45] Fardell JE , Vardy J, Monds LA, Johnston IN. The long-term impact of oxaliplatin chemotherapy on rodent cognition and peripheral neuropathy. Behav Brain Res. 2015;291:80-88. 25934489 10.1016/j.bbr.2015.04.038

[46] Fardell JE , Zhang J, De Souza R, et al The impact of sustained and intermittent docetaxel chemotherapy regimens on cognition and neural morphology in healthy mice. Psychopharmacology (Berl). 2014;231:841-852. 24101158 10.1007/s00213-013-3301-8

[47] Torralva T , Roca M, Gleichgerrcht E, López P, Manes F. INECO frontal screening (IFS): a brief, sensitive, and specific tool to assess executive functions in dementia. J Int Neuropsychol Soc. 2009;15:777-786. http://www.journals.cambridge.org/abstract_S1355617709990415 19635178 10.1017/S1355617709990415

[48] Schilder CMT , Seynaeve C, Linn SC, et al Cognitive functioning of postmenopausal breast cancer patients before adjuvant systemic therapy, and its association with medical and psychological factors. Crit Rev Oncol Hematol. 2010;76:133-141. 20036141 10.1016/j.critrevonc.2009.11.001

[49] Holsinger T , Plassman BL, Stechuchak KM, Burke JR, Coffman CJ, Williams JW. Screening for cognitive impairment: comparing the performance of four instruments in primary care. J Am Geriatr Soc. 2012;60:1027-1036. 22646750 10.1111/j.1532-5415.2012.03967.x

[50] Ahles TA , Li Y, McDonald BC, et al Longitudinal assessment of cognitive changes associated with adjuvant treatment for breast cancer: the impact of APOE and smoking. Psychooncology. 2014;23:1382-1390. 24789331 10.1002/pon.3545 PMC4214914

[51] Shilling V , Jenkins V, Morris R, Deutsch G, Bloomfield D. The effects of adjuvant chemotherapy on cognition in women with breast cancer—preliminary results of an observational longitudinal study. Breast. 2005;14:142-150. 15767184 10.1016/j.breast.2004.10.004

[52] Wefel JS , Vardy J, Ahles T, Schagen SB. International cognition and cancer task force recommendations to harmonise studies of cognitive function in patients with cancer. Lancet Oncol. 2011;12:703-708. 21354373 10.1016/S1470-2045(10)70294-1

[53] Jansen CE , Cooper BA, Dodd MJ, Miaskowski CA. A prospective longitudinal study of chemotherapy-induced cognitive changes in breast cancer patients. Support Care Cancer. 2011;19:1647-1656. http://search.ebscohost.com/login.aspx?direct=true&db=aph&AN=65183089&site=ehost-live 20820813 10.1007/s00520-010-0997-4

[54] Wang XM , Walitt B, Saligan L, Tiwari AFY, Cheung CW, Zhang ZJ. Chemobrain: a critical review and causal hypothesis of link between cytokines and epigenetic reprogramming associated with chemotherapy. Cytokine. 2015;72:86-96. 10.1016/j.cyto.2014.12.006 25573802 PMC4750385

[55] van Dyk K , Ganz PA. Cancer-related cognitive impairment in patients with a history of breast cancer. JAMA. 2021;326:1736-1737. 34652424 10.1001/jama.2021.13309

[56] Franzmeier N , Suárez-Calvet M, Frontzkowski L, et al Higher CSF sTREM2 attenuates ApoE4-related risk for cognitive decline and neurodegeneration. Mol Neurodegener. 2020;15:57-10. 33032659 10.1186/s13024-020-00407-2 PMC7545547

[57] Mazaheri F , Snaidero N, Kleinberger G, et al TREM 2 deficiency impairs chemotaxis and microglial responses to neuronal injury. EMBO Rep. 2017;18:1186-1198. 28483841 10.15252/embr.201743922 PMC5494532

[58] Gonzales HM , Tarraf W, Schneiderman N, et al Prevalence and correlates of mild cognitive impairment among diverse Hispanics/Latinos: study of Latinos-investigation of neurocognitive aging results. Alzheimers Dement. 2019;15:1507-1515. 31753701 10.1016/j.jalz.2019.08.202 PMC7318558

[59] Yang GS , Kumar S, Dorsey SG, Starkweather AR, Kelly DL, Lyon DE. Systematic review of genetic polymorphisms associated with psychoneurological symptoms in breast cancer survivors. Support Care Cancer. 2019;27:351-371. 30343412 10.1007/s00520-018-4508-3

[60] Park JY , Lengacher CA, Reich RR, et al Translational genomic research: the role of genetic polymorphisms in MBSR program among breast cancer survivors (MBSR[BC]). Transl Behav Med. 2019;9:693-702. 30137607 10.1093/tbm/iby061 PMC7184864

[61] Ward DD , Summers MJ, Saunders NL, Ritchie K, Summers JJ, Vickers JC. The BDNF Val66Met polymorphism moderates the relationship between cognitive reserve and executive function. Transl Psychiatry. 2015;5:e590. 10.1038/tp.2015.82 26125153 PMC4490292

[62] Feng LR , Juneau P, Regan JM, et al Brain-derived neurotrophic factor polymorphism Val66Met protects against cancer-related fatigue. Transl Psychiatry. 2020;10:302. 10.1038/s41398-020-00990-4 32848137 PMC7450091

[63] Ohi K , Ursini G, Li M, et al DEGS2 polymorphism associated with cognition in schizophrenia is associated with gene expression in brain. Transl Psychiatry. 2015;5:e550. 25871975 10.1038/tp.2015.45 PMC4462608

[64] Bojar I , Owoc J, Wójcik-Fatla A, Raszewski G, Stančiak J, Raczkiewicz D. Cognitive functions, lipid profile, and apolipoprotein E gene polymorphism in postmenopausal women. Ann Agric Environ Med. 2015;22:313-319. 26094530 10.5604/12321966.1152086

[65] Chae J-W , Ng T, Yeo HL, et al Impact of TNF-α (rs1800629) and IL-6 (rs1800795) polymorphisms on cognitive impairment in Asian breast cancer patients. PLoS One. 2016;11:e0164204. 27701469 10.1371/journal.pone.0164204 PMC5049844

[66] Jiang Y , He T, Deng W, Sun P. Association between apolipoprotein E gene polymorphism and mild cognitive impairment: a meta-analysis. Clin Interv Aging. 2017;12:1941-1949. 29180857 10.2147/CIA.S143632 PMC5691922

[67] Iqbal MUN , Yaqoob T, Ali SA, Khan TA. A functional polymorphism (rs6265, G>A) of brain-derived neurotrophic factor gene and breast cancer: an association study. Breast Cancer (Auckl). 2019;13:1178223419844977. 31105428 10.1177/1178223419844977 PMC6501468

[68] Dooley LN , Ganz PA, Cole SW, Crespi CM, Bower JE. Val66Met BDNF polymorphism as a vulnerability factor for inflammation-associated depressive symptoms in women with breast cancer. J Affect Disord. 2016;197:43-50. Vol 26967918 10.1016/j.jad.2016.02.059 PMC4836957

[69] Tan CJ , Lim SWT, Toh YL, et al Replication and meta-analysis of the association between BDNF Val66Met polymorphism and cognitive impairment in patients receiving chemotherapy. Mol Neurobiol. 2019;56:4741-4750. 30382534 10.1007/s12035-018-1410-4 PMC6647505

[70] Abanmy N , Alsabhan J, Gard P, Scutt G. Association between the Val66Met polymorphism (rs6265/G196A) of the BDNF gene and cognitive performance with SSRI use in Arab Alzheimer’s disease patients. Saudi Pharmaceut J. 2021;29:1392-1398. 10.1016/j.jsps.2021.10.007 PMC8720700 35002376

[71] Cortez-Pacheco R , Acosta O, Casavilca-Zambrano S. Asociación entre el polimorfismo Val66Met del gen BDNF y el desarrollo de disfunción ejecutiva en pacientes con cáncer de mama. Ann Facultad Med. 2023;84:162-167. 10.15381/anales.v84i2.23443

[72] Guio H , Poterico JA, Levano KS, et al Genetics and genomics in Peru: clinical and research perspective. Mol Genet Genomic Med. 2018;6:873-886. 30584990 10.1002/mgg3.533 PMC6305655

[73] Marca V , Acosta O, Cornejo-Olivas M, Ortega O, Huerta D, Mazzetti P. Genetic polymorphism of apolipoprotein e in a Peruvian population. Rev Peru Med Exp Salud Publica. 2011;28:589-594. 22241253

[74] Marca-Ysabel MV , Rajabli F, Cornejo-Olivas M, et al Dissecting the role of amerindian genetic ancestry and the ApoE ε4 allele on Alzheimer disease in an admixed Peruvian population. Neurobiol Aging. 2021;101:298.e11-298.e15. 10.1016/j.neurobiolaging.2020.10.003 PMC8122013 33541779

[75] Chacón-Duque J-C , Adhikari K, Fuentes-Guajardo M, et al Latin Americans show wide-spread converso ancestry and imprint of local native ancestry on physical appearance. Nat Commun. 2018;9:5388. 30568240 10.1038/s41467-018-07748-z PMC6300600

[76] Huerta D , Acosta O, Polo S, Martinez R, Oré R, Miranda C. Polimorfismo Val108/158Met en el gen dopaminérgico catecolo- metil transferasa (COMT) en una población mixta peruana y su importancia para los estudios neuropsiquiátricos. An Fac Med. 2013;68:321-327.

