# Supplementary material for: Cognitive changes associated with chemotherapy in breast cancer: an assessment of social cognition and executive functions in Peruvian patients
Source: Oncologist. 2026 Feb 25;31(4):oyag058. doi: 10.1093/oncolo/oyag058 (PMC12990981; doi:10.1093/oncolo/oyag058)
Supplement: oyag058_Supplementary_Data [file oyag058_supplementary_data.zip › Table S1.docx]

Table S1. Chemotherapy by Molecular Subtype of patients.

| Molecular Subtype | Number of Patients | % of Total | Most Common Treatment | Taxane Initiated | Average Number of Cycles |
| --- | --- | --- | --- | --- | --- |
| Luminal A | 60 | 48.4 | Anthracycline + Alkylating Agent | No | 8 |
| Luminal B | 18 | 14.5 | Anthracycline + Alkylating Agent + Taxane | Yes | 12 |
| Luminal B HER2+ | 6 | 4.8 | Anthracycline + Taxane + Anti-HER2 | Yes | 12 |
| HER2+ | 10 | 8.1 | Taxane + Anti-HER2 | Yes | 12 |
| Triple Negative | 30 | 24.2 | Anthracycline + Taxane | Yes | 12 |
