# Supplementary material for: Cognitive changes associated with chemotherapy in breast cancer: an assessment of social cognition and executive functions in Peruvian patients
Source: Oncologist. 2026 Feb 25;31(4):oyag058. doi: 10.1093/oncolo/oyag058 (PMC12990981; doi:10.1093/oncolo/oyag058)
Supplement: oyag058_Supplementary_Data [file oyag058_supplementary_data.zip › Table S2.docx]

Table S2. Percentage of ancestry of each individual in the study for each superpopulation.

| IID | AFR | NAM | EUR | EAS |
| --- | --- | --- | --- | --- |
| COG_001_MQR | 0.008807 | 0.906193 | 0.084991 | 0.00001 |
| COG_002_CLC | 0.043309 | 0.800644 | 0.156036 | 0.00001 |
| COG_003_JPC | 0.065868 | 0.933769 | 0.000354 | 0.00001 |
| COG_004_GDD | 0.140342 | 0.399133 | 0.460515 | 0.00001 |
| COG_005_JGM | 0.50047 | 0.42439 | 0.07513 | 0.00001 |
| COG_006_MBE | 0.00001 | 0.912496 | 0.087484 | 0.00001 |
| COG_007_ALV | 0.023081 | 0.909101 | 0.067808 | 0.00001 |
| COG_008_RMP | 0.024789 | 0.871846 | 0.101136 | 0.00223 |
| COG_009_EAG | 0.00001 | 0.99997 | 0.00001 | 0.00001 |
| COG_010_GDP | 0.014016 | 0.722507 | 0.257966 | 0.005511 |
| COG_011_BVM | 0.02359 | 0.932607 | 0.038746 | 0.005057 |
| COG_012_VHV | 0.116066 | 0.708476 | 0.125409 | 0.050049 |
| COG_013_GTF | 0.004347 | 0.77601 | 0.219633 | 0.00001 |
| COG_014_VOB | 0.00001 | 0.966862 | 0.033118 | 0.00001 |
| COG_015_RFG | 0.029074 | 0.754796 | 0.21612 | 0.00001 |
| COG_016_MAO | 0.01659 | 0.930624 | 0.052776 | 0.00001 |
| COG_017_LMV | 0.012231 | 0.848175 | 0.12265 | 0.016943 |
| COG_018_DAB | 0.254561 | 0.623883 | 0.113302 | 0.008254 |
| COG_019_MCE | 0.026584 | 0.721802 | 0.242852 | 0.008762 |
| COG_020_BDT | 0.046077 | 0.666171 | 0.286204 | 0.001549 |
| COG_021_JRD | 0.252387 | 0.148749 | 0.598854 | 0.00001 |
| COG_022_YGQ | 0.031908 | 0.847286 | 0.120797 | 0.00001 |
| COG_023_MRR | 0.001977 | 0.849993 | 0.141188 | 0.006842 |
| COG_024_CRT | 0.02982 | 0.700348 | 0.269822 | 0.00001 |
| COG_026_ARR | 0.01125 | 0.98873 | 0.00001 | 0.00001 |
| COG_027_MCH | 0.015417 | 0.852926 | 0.131648 | 0.00001 |
| COG_028_YCV | 0.022014 | 0.671145 | 0.306831 | 0.00001 |
| COG_029_LIM | 0.000018 | 0.999962 | 0.00001 | 0.00001 |
| COG_030_ATG | 0.052852 | 0.591772 | 0.201006 | 0.15437 |
| COG_031_FMQ | 0.02937 | 0.789688 | 0.180931 | 0.00001 |
| COG_032_RPC | 0.00001 | 0.99997 | 0.00001 | 0.00001 |
| COG_033_LPG | 0.013192 | 0.828391 | 0.157194 | 0.001223 |
| COG_034_MVR | 0.040496 | 0.633362 | 0.325263 | 0.000879 |
| COG_035_LRP | 0.072326 | 0.621926 | 0.197138 | 0.10861 |
| COG_036_RMG | 0.356963 | 0.496496 | 0.146531 | 0.00001 |
| COG_038_GGL | 0.017101 | 0.75243 | 0.23046 | 0.00001 |
| COG_039_NDR | 0.022625 | 0.735736 | 0.241629 | 0.00001 |
| COG_040_KGV | 0.02977 | 0.615439 | 0.354781 | 0.00001 |
| COG_041_SAL | 0.038043 | 0.803505 | 0.158442 | 0.00001 |
| COG_043_JCR | 0.031669 | 0.798006 | 0.032048 | 0.138277 |
| COG_044_ARP | 0.00001 | 0.96354 | 0.035197 | 0.001253 |
| COG_045_HMT | 0.367197 | 0.493487 | 0.139306 | 0.00001 |
| COG_046_LPV | 0.023834 | 0.755738 | 0.220418 | 0.00001 |
| COG_047_RCC | 0.189408 | 0.473571 | 0.337011 | 0.00001 |
| COG_048_LCA | 0.022853 | 0.694834 | 0.282303 | 0.00001 |
| COG_049_YCZ | 0.027423 | 0.845509 | 0.127058 | 0.00001 |
| COG_050_CRO | 0.017833 | 0.859935 | 0.122222 | 0.00001 |
| COG_051_GML | 0.00001 | 0.99997 | 0.00001 | 0.00001 |
| COG_052_MMB | 0.27 | 0.264645 | 0.465345 | 0.00001 |
| COG_053_IRA | 0.029284 | 0.787934 | 0.182772 | 0.00001 |
| COG_054_MCL | 0.06204 | 0.89291 | 0.023697 | 0.021353 |
| COG_055_GVH | 0.03182 | 0.795754 | 0.172416 | 0.00001 |
| COG_056_SCP | 0.00001 | 0.99997 | 0.00001 | 0.00001 |
| COG_057_YMP | 0.001048 | 0.932555 | 0.059723 | 0.006675 |
| COG_058_RPF | 0.00001 | 0.974071 | 0.025909 | 0.00001 |
| COG_059_LND | 0.001116 | 0.998864 | 0.00001 | 0.00001 |
| COG_060_MGA | 0.00001 | 0.997947 | 0.00001 | 0.002033 |
| COG_061_DQE | 0.00001 | 0.99997 | 0.00001 | 0.00001 |
| COG_062_SMD | 0.046685 | 0.468231 | 0.481553 | 0.003531 |
| COG_063_ZAY | 0.00001 | 0.828652 | 0.171328 | 0.00001 |
| COG_064_MGP | 0.042218 | 0.750255 | 0.200727 | 0.0068 |
| COG_065_LHQ | 0.122215 | 0.673302 | 0.109674 | 0.094809 |
| COG_066_LVR | 0.00001 | 0.814107 | 0.1827 | 0.003183 |
| COG_067_ZZR | 0.003875 | 0.836836 | 0.145167 | 0.014122 |
| COG_068_AGC | 0.00001 | 0.99997 | 0.00001 | 0.00001 |
| COG_069_SDC | 0.025666 | 0.841068 | 0.133256 | 0.00001 |
| COG_070_JRO | 0.135155 | 0.592322 | 0.269866 | 0.002656 |
| COG_071_ETR | 0.02685 | 0.583323 | 0.385991 | 0.003836 |
| COG_072_MTA | 0.00001 | 0.99997 | 0.00001 | 0.00001 |
| COG_073_CMT | 0.0382 | 0.676757 | 0.271146 | 0.013897 |
| COG_074_OCQ | 0.00001 | 0.99997 | 0.00001 | 0.00001 |
| COG_075_VCG | 0.263822 | 0.488082 | 0.248086 | 0.00001 |
| COG_076_JCC | 0.010868 | 0.871151 | 0.117971 | 0.00001 |
| COG_077_DOL | 0.00001 | 0.99997 | 0.00001 | 0.00001 |
| COG_078_RMC | 0.00001 | 0.952374 | 0.041633 | 0.005983 |
| COG_079_MCM | 0.041779 | 0.592203 | 0.366008 | 0.00001 |
| COG_080_ECC | 0.053916 | 0.797848 | 0.147432 | 0.000804 |
| COG_081_ICS | 0.02271 | 0.705773 | 0.271506 | 0.00001 |
| COG_082_MRE | 0.198517 | 0.185164 | 0.616309 | 0.00001 |
| COG_083_LMC | 0.011292 | 0.663349 | 0.319826 | 0.005533 |
| COG_084_JPP | 0.00001 | 0.964254 | 0.027234 | 0.008502 |
| COG_085_ARI | 0.467339 | 0.311086 | 0.221565 | 0.00001 |
| COG_086_LIF | 0.00001 | 0.837532 | 0.154997 | 0.007462 |
| COG_087_VRR | 0.228194 | 0.550462 | 0.221335 | 0.00001 |
| COG_088_MCG | 0.028651 | 0.725887 | 0.236471 | 0.008992 |
| COG_089_RVA | 0.011278 | 0.904338 | 0.069178 | 0.015206 |
| COG_090_MAD | 0.201724 | 0.59561 | 0.202656 | 0.00001 |
| COG_091_JLG | 0.043373 | 0.57846 | 0.378157 | 0.00001 |
| COG_092_BGC | 0.138592 | 0.561198 | 0.3002 | 0.00001 |
| COG_093_JFB | 0.00001 | 0.973297 | 0.026683 | 0.00001 |
| COG_094_YCD | 0.140868 | 0.742764 | 0.116359 | 0.00001 |
| COG_095_VGC | 0.012948 | 0.791288 | 0.195753 | 0.00001 |
| COG_096_EVS | 0.00001 | 0.862761 | 0.135593 | 0.001636 |
| COG_097_YMV | 0.00001 | 0.969686 | 0.030294 | 0.00001 |
| COG_098_DHZ | 0.003377 | 0.791786 | 0.204828 | 0.00001 |
| COG_099_CLB | 0.000266 | 0.880306 | 0.115366 | 0.004061 |
| COG_100_FCL | 0.00001 | 0.99997 | 0.00001 | 0.00001 |
| COG_101_VVR | 0.112406 | 0.619293 | 0.268011 | 0.00029 |
| COG_102_GVT | 0.17721 | 0.694765 | 0.128016 | 0.00001 |
| COG_103_NQF | 0.021307 | 0.930503 | 0.048181 | 0.00001 |
| COG_104_PFC | 0.051598 | 0.842204 | 0.106188 | 0.00001 |
| COG_105_RVC | 0.001869 | 0.998111 | 0.00001 | 0.00001 |
| COG_106_LOC | 0.000635 | 0.779664 | 0.219269 | 0.000432 |
| COG_107_APA | 0.029761 | 0.567251 | 0.402978 | 0.00001 |
| COG_108_CAJ | 0.00001 | 0.99997 | 0.00001 | 0.00001 |
| COG_109_APS | 0.00001 | 0.99997 | 0.00001 | 0.00001 |
| COG_110_JVC | 0.015076 | 0.858672 | 0.126242 | 0.00001 |
| COG_111_NRR | 0.00001 | 0.866398 | 0.125357 | 0.008235 |
| COG_112_VCA | 0.00001 | 0.856186 | 0.127593 | 0.016212 |
| COG_113_PSH | 0.024975 | 0.883452 | 0.091563 | 0.00001 |
| COG_114_RVC | 0.067414 | 0.630327 | 0.223836 | 0.078423 |
| COG_115_IOP | 0.047189 | 0.869299 | 0.081102 | 0.00241 |
| COG_116_MLA | 0.215112 | 0.505441 | 0.279436 | 0.00001 |
| COG_117_STP | 0.141533 | 0.60489 | 0.253567 | 0.00001 |
| COG_118_JMM | 0.033118 | 0.818125 | 0.148748 | 0.00001 |
| COG_119_PGI | 0.00001 | 0.99997 | 0.00001 | 0.00001 |
| COG_120_MRM | 0.244388 | 0.630783 | 0.124819 | 0.00001 |
| COG_121_ZHM | 0.000466 | 0.904325 | 0.093696 | 0.001513 |
| COG_122_LRA | 0.00001 | 0.921246 | 0.078734 | 0.00001 |
| COG_123_MCS | 0.043754 | 0.660819 | 0.295416 | 0.00001 |
| COG_124_RQA | 0.035117 | 0.784393 | 0.136429 | 0.044061 |
| COG_125_IAD | 0.00001 | 0.99997 | 0.00001 | 0.00001 |
| COG_126_AVN | 0.069294 | 0.763362 | 0.167334 | 0.00001 |
| COG_127_MLT | 0.221764 | 0.570722 | 0.207504 | 0.00001 |
| COG_128_EPR | 0.152284 | 0.630629 | 0.217076 | 0.00001 |
| COG_129_CPA | 0.00001 | 0.944326 | 0.040239 | 0.015425 |
| COG_130_GCA | 0.00001 | 0.989396 | 0.009563 | 0.001031 |
| COG_131_JFM | 0.086331 | 0.778556 | 0.133119 | 0.001994 |
| COG_132_LAH | 0.039733 | 0.767524 | 0.192733 | 0.00001 |
| COG_133_RTC | 0.068316 | 0.705855 | 0.22582 | 0.00001 |
| COG_134_AGA | 0.007328 | 0.882786 | 0.108509 | 0.001378 |
| COG_135_TJV | 0.00001 | 0.981451 | 0.018529 | 0.00001 |
| COG_136-MLR | 0.027466 | 0.471604 | 0.490118 | 0.010812 |
| COG_137-MMV | 0.038672 | 0.821346 | 0.134403 | 0.00558 |
| COG_138_NZY | 0.046324 | 0.827562 | 0.110892 | 0.015222 |
| mean | 0.05780744 | 0.7776965 | 0.15749799 | 0.00699816 |
| SD | 0.09299216 | 0.18350995 | 0.12948694 | 0.02297696 |

Note: The populations were downloaded from the 1000 Genomes Project database and analysed with RStudio. IID: Individual ID; Superpopulations, AFR, African; NAM, American; EUR, European; EAS, East Asian; SD, Standard deviation
